# Associations between Verbal Fluency and Asymmetry of White Matter Integrity in the Superior Longitudinal Fasciculus in At-Risk Mental States for Psychosis

**DOI:** 10.3390/jpm14030228

**Published:** 2024-02-21

**Authors:** Junichi Saito, Naoyuki Katagiri, Hiromi Tagata, Yu Arai, Kouhei Kamiya, Masaaki Hori, Masafumi Mizuno, Takahiro Nemoto

**Affiliations:** 1 Department of Neuropsychiatry, Toho University Faculty of Medicine, 6-11-1 Omori-nishi, Ota-ku, Tokyo 143-8541, Japan; junichi.saitou@med.toho-u.ac.jp (J.S.); ktgrnoyk@med.toho-u.ac.jp (N.K.); hiromi.tagata@med.toho-u.ac.jp (H.T.); yu.arai@med.toho-u.ac.jp (Y.A.); masafumi_mizuno@tmhp.jp (M.M.); 2 Department of Neuropsychiatry, Toho University Graduate School of Medicine, 5-21-16 Omori-nishi, Ota-ku, Tokyo 143-8540, Japan; 3 Department of Radiology, Toho University Omori Medical Center, 6-11-1 Omori-nishi, Ota-ku, Tokyo 143-8541, Japan; kohei.kamiya@med.toho-u.ac.jp (K.K.); masahori@med.toho-u.ac.jp (M.H.); 4 Tokyo Metropolitan Matsuzawa Hospital, 2-1-1 Kamikitazawa, Setagaya-ku, Tokyo 156-0057, Japan; 5 Department of Psychiatry and Implementation Science, Toho University Faculty of Medicine, 6-11-1 Omori-nishi, Ota-ku, Tokyo 143-8541, Japan

**Keywords:** at-risk mental states, early intervention, laterality index, tractography, verbal fluency

## Abstract

Verbal fluency is one of the most severely impaired components of cognitive function in schizophrenia and is also impaired in at-risk mental states (ARMSs) for psychosis. The aim of this study was to explore the markers of disease progression in subjects with ARMSs by comparing the association between the white matter integrity of the superior longitudinal fasciculus (SLF) and verbal fluency in subjects with ARMSs and healthy control (HC) subjects. The correlations of the fractional anisotropy (FA) values on diffusion tensor imaging (DTI) and the laterality index (LI) values of SLF branches I, II, and III with the verbal fluency performance were analyzed in right-handed subjects with ARMSs (ARMS group; *n* = 18) and HC subjects (HC group; *n* = 34) aged 18 to 40 years old. In the HC group compared with the ARMS group, the LI values suggested right lateralization of the SLF II and III. Letter fluency was significantly correlated with the LI of the SLF III in both the ARMS and HC groups. The regression coefficient (β) of this correlation was calculated using the least squares method and yielded a positive number (73.857) in the ARMS group and a negative number (−125.304) in the HC group. The association of the rightward asymmetry of the SLF III with the verbal fluency performance observed in the HC group appeared to be lost in the ARMS group, and this could serve as one of the markers of the pathological progression to psychosis in patients with schizophrenia.

## 1. Introduction

Patients with schizophrenia present with a variety of cognitive deficits. Verbal fluency is one of the most severely impaired cognitive functions in patients with schizophrenia [1,2]. Verbal fluency tasks involve recalling vocabulary while remembering the task rules and continuing word retrieval while avoiding vocabulary that has already been used. Therefore, verbal fluency tasks require the use of short-term memory and working memory [3].

The superior longitudinal fasciculus (SLF) is considered an important component of the white matter involved in cognitive function and language. The SLF comprises a large bundle of medial and lateral fibers connecting the frontal, parietal, and temporal lobes [4]. These white matter fiber connections comprise three subcomponents that originate in the caudal cortex and connect to different parts of the temporal-parietal junction, with the SLF II terminating in the dorsolateral prefrontal cortex and the SLF III terminating in the ventrolateral prefrontal cortex. The SLF II and III tend to be larger in the right hemisphere than in the left [5].

In terms of the white matter integrity asymmetry, diffusion tensor imaging (DTI) studies have reported a rightward asymmetry of the fractional anisotropy (FA) values for the SLF III in healthy control (HC) subjects and in patients with schizophrenia [6]. However, only HC subjects had this pattern regarding the SLF II [6]. The SLF is a large white matter fiber bundle connecting extensive regions of the frontal and parietal lobes. The notable asymmetry in these white matter fibers suggests that the morphological differences are likely reflected in functional significance. It has been suggested that the attentional functions required for verbal fluency derive primarily from the hemispheric asymmetries in the frontoparietal fiber network comprised of the SLF segments I, II, and III [5]. The SLF III is suggested to play a role in speech in the left hemisphere and in spatial recognition in the right hemisphere [7]. The SLF II is presumed to be involved in the motor planning of speech and syntactic processing in the left hemisphere, while in the right hemisphere, faster and preferential visuospatial processing and regulating attention to spatial orientation occurs [7].

Verbal fluency can be divided into two main categories: letter fluency and category fluency. Letter fluency involves producing words based on phonemic features, such as initial letters and sounds. Category fluency requires the production of words that belong to a defined semantic category [8]. A meta-analysis reported that category fluency and letter fluency are impaired in patients with schizophrenia [9]. A lack of verbal fluency is considered a predictor of psychosis, whereas, in patients with chronic schizophrenia, the symptoms are fixed [10]. In another study, a category fluency task was administered to adolescent patients with schizophrenia and was found to be significantly correlated with other cognitive functions and psychiatric symptoms, particularly those related to verbal learning, working memory, and processing speed. In addition, moderate correlations were found with total positive and negative syndrome scale (PANSS) scores and with negative, positive, and cognitive PANSS factors. It was suggested that the analysis of category fluency components may provide information on cognitive function and psychiatric symptom profiles in adolescent patients with schizophrenia [11].

Although there is still debate as to whether deficits in category fluency or letter fluency deficits are more serious in schizophrenia, a previous study using functional near-infrared spectroscopy indicated that patients with schizophrenia showed reduced left lateralization of cerebral activity during a category fluency task, which may be associated with impaired semantic abilities [10]. In a functional magnetic resonance imaging (fMRI) study focusing on patients with first-episode schizophrenia (FES) who were not receiving pharmacological treatment, the research aimed to investigate the impact of antipsychotic medication on the abnormal lateralization of verbal-related functions and changes in cognitive function and psychiatric symptoms. Following antipsychotic treatment, a significant increase in the lateralization index of the inferior frontal gyrus was observed. Importantly, this heightened lateralization index was found to be significantly associated with improvements in verbal fluency scores. Notably, individuals with superior baseline performance on the category fluency tasks demonstrated elevated lateralization indices in the inferior frontal gyrus post-treatment. Additionally, substantial improvements in the PANSS total scores and positive subscores were noted in these individuals. These findings suggest that antipsychotic treatment in individuals not receiving medication for FES can significantly enhance lateralization in the inferior frontal gyrus, particularly in relation to semantic processing. This sheds light on the potential positive impact of antipsychotic interventions on both cognitive function and psychiatric symptomatology in this specific population [12]. These findings lend support to the hypothesis that impaired category fluency performance might be a characteristic marker of early schizophrenia and could be the result of a failure of the lateralization of cerebral verbal function.

DTI studies have been conducted to identify the anatomical correlates of impaired verbal fluency in patients with schizophrenia. It was reported that the FA values of the right SLF are lower in patients with FES than in HC subjects [13]. Mega-analyses have shown that the FA values of white matter, including the SLF, are lower in adolescents with early-onset schizophrenia than in HC subjects [14]. The SLF is involved in cognitive functions, such as working memory, attention, and verbal processing, and this suggests that the SLF may be involved in the pathogenesis of psychosis [14]. In addition, patients have also been examined for their social cognitive functions, including verbal learning and speed of processing, and positive correlations of the scores with the FA values of the right SLF were reported [13]. The performance in category fluency and attention tests in young patients with psychosis was reported to be correlated with the FA values of the left SLF [15]. These reports indicate that white matter integrity, in particular, of the left and/or right SLF, determines the cognitive prognosis in patients with schizophrenia and, further, that the structural asymmetry of the SLF segments between the left and right sides may also be related to the cognitive functions of these patients.

Verbal fluency is also impaired in subjects with at-risk mental states (ARMSs) for psychosis. Category fluency was significantly inferior in both subjects with ARMSs and patients with FES as compared with that in HC subjects [12]. In patients with schizophrenia, noticeable alterations in verbal-related functions often manifest. A hallmark symptom of schizophrenia is characterized as “poverty of speech”, encompassing diminished category fluency, impaired word and sentence generation, impaired processing of complex syntax, and pragmatic language impairments. Additionally, in subjects with ARMSs, manifestations such as “poverty of content”, pragmatic difficulties, and reduced verbal fluency have been observed. These changes may carry predictive significance, hinting at an elevated probability of the future development of schizophrenia [16]. A comparison of category fluency performance between subjects with ARMSs and HC subjects revealed that category fluency was worse in the ARMS group. On the other hand, there was a lack of consensus regarding letter fluency [16]. In a previous study, patients with ARMSs, FES, and schizophrenia relapse (the psychosis group) showed worse performance on category fluency and attention compared with HC subjects [17]. Category fluency and spatial working memory performance in the ARMS group were intermediate between those in the patients with FES and HC subjects [18]. The degree of leftward asymmetry reduction of the FA values of the cingulate bundle in subjects with ARMSs may determine their decline in social functioning [19]. To the best of our knowledge, there is only one published report in the literature that has verified the asymmetry of segments of the SLF in subjects with ARMSs. The rightward asymmetry of the FA values of the SLF III was larger in women in the ARMS group than in HC subjects. Furthermore, greater rightward asymmetry of the SLF III was reported as being associated with reduced working memory function [20].

The aim of this study was to investigate disease progression in subjects with ARMSs by comparing the association of the white matter integrity, in particular, the SLF, with verbal fluency in subjects with ARMSs and HC subjects.

## 2. Materials and Methods

### 2.1. Participants

Individuals aged between 18 and 40 years at the time of their initial visit to the Mental Health Center at Toho University Omori Medical Center, Tokyo, were asked to complete the prevention through risk identification, management, and education (PRIME) screen-revised questionnaire for identifying ARMS candidates [21]. After this screening, the subjects were screened using the structured interview for prodromal symptoms/the scale of prodromal symptoms (SIPSs/SOPSs) to confirm the diagnosis of ARMS [21]. HC subjects between 18 and 40 years of age were recruited from the general population. The identification of healthy control subjects was performed strictly according to the Japanese version [22] of the structured clinical interview for DSM-IV-TR axis I disorders (SCID-IV) non-patient edition [23].

The subjects with ARMSs (ARMS group) and HC subjects (HC group) were administered the verbal fluency test and underwent magnetic resonance imaging (MRI) of the brain. Only right-handed individuals, identified by performing the Edinburgh handedness inventory [24], were included in both the ARMS group and the HC group. The exclusion criteria for the study included a history of neurological disorders and/or alcohol/substance abuse.

### 2.2. MRI Acquisition

MRI was performed using a 1.5-T scanner (Signa HDxt, GE Medical Systems, Waukesha, WI, USA). An 8-channel NV HEAD coil was used to receive the MRI signal. Diffusion tensor analysis was also performed (repetition time/echo time = 13,000/76.8 ms; 3 mm slice thickness with no gap; field of view [FOV] = 240 × 240 mm; number of excitations = 1; 80 × 80-pixel matrix; b = 1000 s/mm^2^; acquisition time = 6.56 min). Images were obtained with both 30-direction diffusion encoding (b = 1000 s/mm^2^ for each direction) and no diffusion encoding (b = 0 s/mm^2^).

### 2.3. Image Analysis

The raw images were denoised and corrected for Gibbs ringing. Then, the correction for eddy current and motion was performed using the eddy tool in the FMRIB software library (FSL Version 6.0.4). Finally, the images were minimally smoothed with a Gaussian kernel with sigma = 1 mm to suppress the effects of residual noise and Gibbs artifacts. A DTI FA map was computed using the standard weighted least square fit implemented in MRTrix3 Version 3.0.2. The segmentation of the white matter tracts was performed using TractSeg, which allows for the semi-automatic reconstruction of the fiber bundles in the subject’s native space. The tracts were extracted from the SLF I, II, and III of the right and left sides, and the FA values were analyzed.

### 2.4. Evaluation of Psychiatric Symptoms

The ARMS group completed the SIPSs/SOPSs screening. The SOPSs scale consists of five items for positive symptoms (P-score), six items for negative symptoms (N-score), four items for disorganization symptoms (D-score), and four items for general symptoms (G-score). There are seven anchor points, from 0 (never or absent) to 6 (severe or extreme) for each of the items. Diagnosis of the prodromal status proceeded as follows: P-scores of 0 to 2 on the SOPSs scale indicated a non-prodromal status, P-scores of 3 to 5 indicated a prodromal status, and a P-score of 6 indicated a psychotic state [21].

### 2.5. Evaluation of Verbal Fluency

In the letter fluency test, the participants were asked to say as many words as possible beginning with a given kana (syllables), “shi”, “i”, and “re” within 60 s, excluding proper nouns, numbers, and the same words with different suffixes [8]. The three kanas appear at different frequencies in the Japanese vocabulary. High-frequency words beginning with the characters “shi” and “i” as well as low-frequency words beginning with “re” were selected from Japanese word frequencies.

In the Japanese version of the category fluency test, which has been shown to be highly reliable, the participants were asked to name as many animals, fruits, and vehicles as possible within 60 s [8].

### 2.6. Evaluation of Brain Structural Asymmetry

We used the laterality index (LI) calculated from the DTI study, which is defined as the ratio of the FA values ([left − right]/[left + right]), to evaluate the structural asymmetry of the brain [25]. The DTI-based LI values were calculated for the bilateral SLF I, II, and III. The LI is commonly used to evaluate brain structural asymmetry and provides values between −1 and +1. Negative and positive values reflect a rightward and a leftward laterality, respectively [25].

### 2.7. Statistical Analysis of the Verbal Fluency Scores and Imaging Data

The statistical analysis was performed using the SPSS 22.0 software. In cross-sectional studies, the Mann-Whitney U-test was used to compare the letter and category fluency scores, the FA values of the bilateral SLF I, II, and III, and the LI values of the SLF I, II, and III between the ARMS group and HC group. 

For the mean FA values of the SLF I, II, and III of both sides and the LI values of the SLF I, II, and III that differed significantly between the ARMS group and HC group, correlations with the letter and category fluency scores in the two groups were analyzed by determining the Spearman rank correlations. The significance level for the Spearman rank correlations was set at *p* < 0.05.

For each of the correlated results, a scatter plot was drawn, and the regression coefficient (β) of the line was determined using the least squares method.

### 2.8. Ethical Standards

The study was conducted with the approval of the Ethics Committee of Toho University Faculty of Medicine (Application No. A19078 and A20026) and in compliance with the principles of the Declaration of Helsinki. All the participants and their parents (for patients who were minors) provided written informed consent for participation in the study.

## 3. Results

### 3.1. Demographic Data

The demographic data of the subject population for this study are shown in Table 1. There was a total of 18 subjects in the ARMS group, including 7 men and 11 women, with a mean age of 23.667 ± 4.875 years (mean ± S.D.) and a mean number of years of education of 12.056 ± 1.589 years. In the HC group, there were a total of 34 subjects, including 21 men and 13 women, with a mean age of 25.971 ± 4.933 years and a mean number of years of education of 15.735 ± 1.620 years.

### 3.2. Verbal Fluency

As shown in Table 2, the letter fluency and category fluency scores were 26.222 ± 6.074 and 41.500 ± 7.382, respectively, in the ARMS group and 29.676 ± 7.446 and 45.618 ± 7.758, respectively, in the HC group. The category fluency score was clearly higher than the letter fluency score in both the ARMS group and the HC group. While the differences were not statistically significant, both the mean letter fluency and category fluency scores tended to be higher in the HC group than in the ARMS group.

### 3.3. Tractography

**Figure 1:**
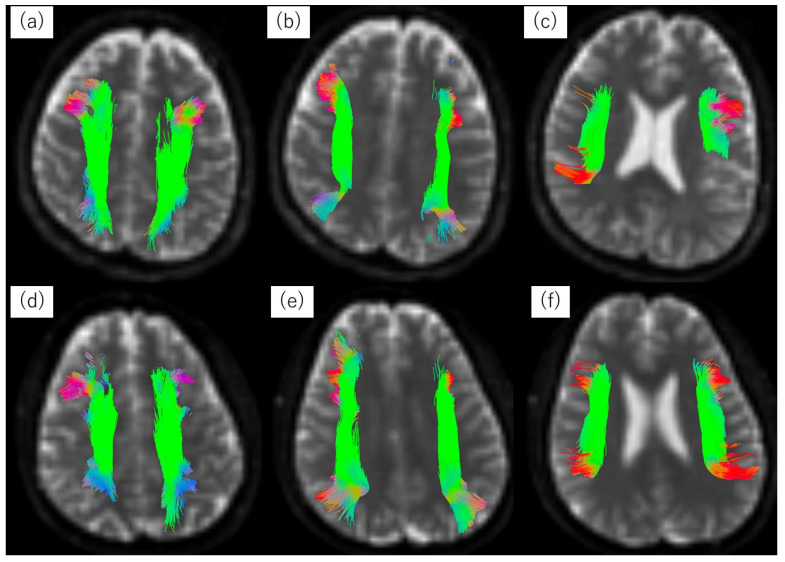
Tractography of the superior longitudinal fasciculus (SLF) I, II, and III of the right and left sides in the at-risk mental state (ARMS) group and the healthy control (HC) group. In the color maps, the colors red, green, and blue were assigned to the left–right, anteroposterior, and craniocaudal directions, respectively. (**a**) Tractography of the SLF I, (**b**) SLF II, and (**c**) SLF III in the ARMS group; (**d**) Tractography of the SLF I, (**e**) SLF II, and (**f**) SLF III in the HC group.

The tractography of the SLF I, II, and III of the right and left sides (Figure 1a: SLF I, Figure 1b: SLF II, and Figure 1c: SLF III in the ARMS group; Figure 1d: SLF I, Figure 1e: SLF II, and Figure 1f: SLF III in the HC group) revealed no significant differences in the FA values between the ARMS group and the HC group (Table 2). The LI values suggested the right lateralization of the SLF II and III in the HC group compared with the ARMS group (Table 2).

### 3.4. Correlations between the Verbal Fluency and LI of the SLF

**Figure 2:**
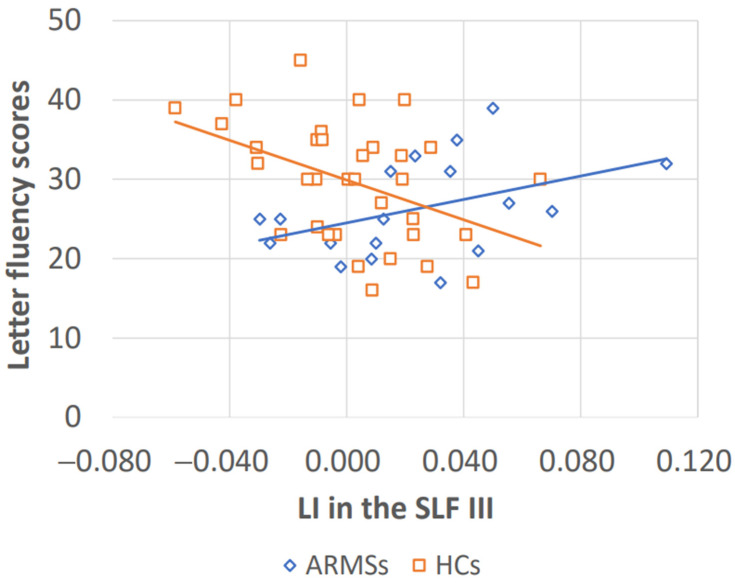
Regression coefficient (β) of the scatter plots of the laterality index (LI) in the superior longitudinal fasciculus (SLF) III and letter fluency scores in subjects with at-risk mental states (ARMSs) and healthy control (HC) subjects. The regression coefficient (β) of this correlation was calculated using the least squares method and was a positive number (73.857) for the ARMS group (blue line) and a negative number (−125.304) for HCs (orange line).

Correlations between the verbal fluency scores and the LI values of the SLF II and III in both the ARMS group and HC group were examined. As shown in Table 3, the letter fluency score was significantly correlated with the LI value of the SLF III in both the ARMS group (r, *p*) = (0.498, 0.036) and the HC group (−0.437, 0.010). The regression coefficient (β) of this correlation was calculated using the least squares method and was a positive number (73.857, leftward laterality) in the ARMS group and a negative number (−125.304, rightward laterality) in the HC group (Figure 2).

## 4. Discussion

Based on the concept that ARMS is an intermediate stage in the disease progression from a healthy state to schizophrenia, this study was a “quantitative study” to capture and explore indicators of the morphological changes in the brain at each stage of the disease. As a study to explore the predictors of disease onset and prognosis in patients with schizophrenia, this study focused on the SLF as a candidate region involved in verbal fluency. The regression coefficient (β) of this correlation was calculated using the least squares method and was a positive number (73.857) in the ARMS group and a negative number (−125.304) in the HC group. The association of the rightward asymmetry of the SLF III with verbal fluency performance observed in the HC group appeared to have been lost in the ARMS group, and this could reflect the progression of the pathological process toward psychosis. These findings seem to provide useful insight for exploring biomarkers to predict disease onset and prognosis in patients with schizophrenia.

The following recent previous studies support our results regarding the laterality of the SLF. One study reported that the SLF II and III showed rightward FA asymmetry in healthy adolescents and adults [6]. In typically developing children, better scores on category fluency and letter fluency were associated with higher FA values of the right SLF/arcuate fasciculus, suggesting that children’s verbal fluency is dependent on the structure of the right hemisphere [26]. In a comparison between HC subjects and patients with schizophrenia, the HC subjects showed higher white matter integrity in the right SLF II and SLF III than the patients with schizophrenia. A comparison of the asymmetry of the white matter integrity revealed that while the SLF III showed greater integrity on the right side than on the left side in both groups, this tendency for the SLF II was only observed in the HC group. Patients with schizophrenia showed a loss of white matter asymmetry of the SLF II [6]. It was reported that the FA values of the left SLF are lower in schizophrenia patients with early-stage illness than in normal subjects [27]. In the mega-analysis, the most prominent effect on the reduced FA values in early-onset schizophrenia during adolescence was observed in the SLF [14]. This finding stands in contrast to the effects reported in adults, where the most significant impacts were noted in the anterior thalamic radiation and the corpus callosum. The distinct variations in the SLF were more conspicuous in the early-onset group [14]. The SLF is involved in working memory, attention, and verbal and emotional processing [14]. Significant deficits in the SLF white matter microstructure were also reported in people with ARMSs, early-onset schizophrenia, and schizophrenia [14]. These findings suggest that the SLF may be involved in the development of psychosis.

In addition, the study suggests that abnormalities in the neurodevelopmental maturation of the SLF may have an influence on psychiatric symptoms. Patients with schizophrenia showed abnormalities in SLF II and SLF III integrity compared to HC subjects, suggesting neuronal loss and fiber demyelination [6]. This study revealed differences in white matter integrity between patients with schizophrenia and HC subjects with respect to anatomical organization, corresponding to visuospatial abilities [6]. In particular, they consider that the asymmetrical difference between the two groups in the SLF II with respect to white matter integrity, i.e., the reduction in white matter integrity observed in patients with schizophrenia, was more prominent in the SLF II and was associated with both axonal and myelin sheath reduction [6]. In healthy subjects aged 8–21 years old, the FA values in the SLF of both sides were positively correlated with verbal working memory. This region was shown to contribute to increased verbal fluency with increasing age, suggesting that the abnormal neurodevelopmental maturation of the SLF during adolescence is an important indicator of schizophrenia [28]. The examination of the relationship between verbal fluency and SLF integrity from the perspective of language acquisition showed that the SLF III was associated with a higher articulation rate in healthy native English speakers acquiring French as a second language [29]. Some reports have also suggested that the severity of the psychiatric symptoms and cognitive deficits in schizophrenia is not correlated with the degeneration of specific areas of the cerebral white matter but is rather associated with defects in the white matter pathways [30]. This suggests that the SLF and the circuit systems containing the SLF may degenerate in subjects with ARMSs and patients with schizophrenia and/or may develop via different mechanisms in patients with ARMSs and schizophrenia than in HC subjects.

### Limitations

Firstly, all participants in this study were native Japanese speakers. It was pointed out that a subject’s native language and culture influence their word production strategies in verbal fluency tasks [31]. Recent neuroimaging studies have also shown that even HC subjects show language-dependent differences in the patterns of brain activity when performing cognitive tasks [32]. On the other hand, it was reported that patients with schizophrenia whose native language is Japanese are equally impaired in the execution of category fluency and letter fluency, unlike patients who speak alphabetic languages [33]. To the best of our knowledge, however, this is the first study to analyze the relationship between verbal fluency and white matter integrity in the SLF III in subjects with ARMSs. We believe that such a detailed study of the difference in the relationship between impaired verbal fluency and white matter integrity between healthy subjects and subjects with ARMSs would be very useful to examine the prognosis of individuals who may develop psychosis and to use this information for early clinical interventions.

A second limitation was the small sample size of the ARMS group. This was due to the presence of several ARMS subjects with missing data on the SOPSs score who could not be included in the analysis. However, it is significant that we were able to point out the importance of qualitative research by collecting and analyzing data on verbal fluency and DTI in addition to SOPSs in subjects with ARMSs.

The third limitation of the study was our use of MRI with a 1.5-T scanner for DTI acquisition. Because the MRI equipment that we used was intended for clinical applications and not for research purposes, our data were, unfortunately, not collected using a 3.0-T or higher equipment. A 3.0-T can achieve a higher b-value and signal-to-noise ratio (SNR) than a 1.5-T, and a higher b-value and SNR improve the FA contrast and tractography in DTI because of better resolution of commissural fibers [34]. The greatest advantage of a 3.0-T MRI is that its SNR is up to twice that of a 1.5-T. Therefore, it enables imaging with higher in-plane resolution and thinner slice thickness than a 1.5-T. Therefore, caution is needed in interpreting the present results; nevertheless, we believe that our results regarding the relationship between the DTI parameters and verbal fluency will serve as useful information for studies on biomarkers of disease onset and prognosis in patients with schizophrenia.

Finally, the SCID was administered to HC subjects. However, the SOPSs test was not conducted on them. Given that subclinical cases of mental disorders may exist in the general population, more thorough exclusion may have been necessary.

In the present study, the ARMS group had a higher proportion of females compared to the HC group. Although it is necessary to consider the possibility of gender imbalance acting as a confounding factor in the analysis of the SLF, we deem this study as a preliminary exploring study and need further analysis using larger samples in the future.

## 5. Conclusions

The association of the rightward asymmetry of the SLF III with verbal fluency observed in healthy subjects appears to be lost in individuals with ARMSs. This structural change in white matter may reflect the progression of the pathological process in patients with psychotic disorders.

## Figures and Tables

**Table 1:** Demographic and clinical characteristics of the participants.

	At-Risk Mental States (ARMSs)	Healthy Controls (HCs)
Male/Female	7/11	21/13
Age (years)	23.667 ± 4.875	25.971 ± 4.933
Education (years)	12.056 ± 1.589	15.735 ± 1.620
SOPSs P-score	14.111 ± 3.563	
SOPSs N-score	14.056 ± 5.683	
SOPSs D-score	5.111 ± 3.324	
SOPSs G-score	8.556 ± 4.003	

The values are means ± SD unless otherwise noted. SOPSs = the scale of prodromal symptoms, P-score = positive symptom score, N-score = negative symptom score, D-score = disorganization symptom score, G-score = general symptom score.

**Table 2:** Verbal fluency scores, fractional anisotropy values, and laterality indices of the superior longitudinal fasciculus I, II, and III in the participants.

		At-Risk Mental States (ARMSs) *n* = 18	Healthy Controls (HCs) *n* = 34	Z	*p*
Verbal fluency scores	Letter fluency	26.222 ± 6.074	29.676 ± 7.446	−1.619	0.105
	Category fluency	41.500 ± 7.382	45.618 ± 7.758	−1.521	0.128
Fractional anisotropy (FA) values	SLF I (left)	0.374 ± 0.015	0.373 ± 0.017	−0.058	0.954
	SLF I (right)	0.368 ± 0.020	0.371 ± 0.015	−0.731	0.465
	SLF II (left)	0.325 ± 0.019	0.319 ± 0.031	−0.212	0.832
	SLF II (right)	0.331 ± 0.020	0.339 ± 0.019	−1.558	0.119
	SLF III (left)	0.383 ± 0.035	0.377 ± 0.016	−0.404	0.686
	SLF III (right)	0.365 ± 0.023	0.376 ± 0.015	−1.577	0.115
Laterality indices (LI)	SLF I	0.008 ± 0.023	0.003 ± 0.026	−0.692	0.489
	SLF II	−0.010 ± 0.024	−0.032 ± 0.056	−2.097	0.036
	SLF III	0.023 ± 0.036	0.002 ± 0.026	−2.231	0.026

The values are means ± SD unless otherwise noted. SLF = superior longitudinal fasciculus.

**Table 3:** Correlations between the verbal fluency scores and laterality index values of the superior longitudinal fasciculus II and III in the participants.

Verbal Fluency Scores	At-Risk Mental States (ARMSs) *n* = 18		Healthy Controls (HCs) *n* = 34	
	Letter Fluency	Category Fluency	Letter Fluency	Category Fluency
	r, *p*	r, *p*	r, *p*	r, *p*
LI in the SLF II	0.243, 0.332	0.248, 0.321	0.217, 0.217	0.126, 0.477
LI in the SLF III	0.498, 0.036	0.094, 0.710	−0.437, 0.010	−0.285, 0.102

The values are means ± SD unless otherwise noted. SLF = superior longitudinal fasciculus, LI = laterality index.

## Data Availability

The data that served as the basis for the findings of the present study are available upon request from the corresponding author. The data are not publicly available due to privacy/ethical restrictions.
